# A comparative study of sociodemographic characteristics, health cognition, and risky sexual behaviors between MSMO and MSMW in Zhejiang Province, Eastern China

**DOI:** 10.3389/fpubh.2026.1824375

**Published:** 2026-04-29

**Authors:** Lin He, Guoying Zhu, Ying Zhu, Tingting Jiang, YangYang Tian, Rui Ge, Chengliang Chai

**Affiliations:** 1 Zhejiang Provincial Center for Disease Control and Prevention, Hangzhou, Zhejiang, China; 2 Jiaxing Center for Disease Control and Prevention, Jiaxing, Zhejiang, China; 3 Jiashan County Center for Disease Control and Prevention, Jiashan, Zhejiang, China

**Keywords:** cross-sectional study, health cognition, HIV risk behaviors, men who have sex with men and women, sexual health

## Abstract

**Background:**

Men who have sex with men (MSM) are a high-risk group for HIV, with marked heterogeneity in sexual behaviors. However, MSM are often treated as a homogeneous population, limiting the effectiveness of targeted HIV prevention strategies. This study aimed to compare sociodemographic characteristics, health cognition, and sexual risk behaviors between men who have sex with men only (MSMO) and men who have sex with men and women (MSMW) in Zhejiang Province, Eastern China.

**Methods:**

This cross-sectional study was conducted among MSM in Zhejiang Province between July and December 2023. Participants were recruited through online channels and convenience sampling via non-governmental organizations (NGOs), and completed an anonymous online questionnaire. Bivariate analyses and multivariable logistic regression were used to identify factors associated with MSMW compared with MSMO.

**Results:**

A total of 7,629 participants were included, comprising 5,964 MSMO and 1,665 MSMW. Compared with MSMO, MSMW were more likely to be married or cohabiting (adjusted odds ratio [aOR]: 2.38, 95% Confidence Interval [CI]: 1.68–3.37), report first-time HIV testing (aOR: 1.62, 95% CI: 1.36–1.92), and identify as having versatile (aOR: 9.56, 95% CI: 7.51–12.16) or insertive (aOR: 3.87, 95% CI: 3.01–4.99) sexual roles. MSMW also demonstrated significantly lower awareness of post-exposure prophylaxis (PEP) (aOR: 0.77, 95% CI: 0.64–0.94) and monkypox (MPOX) (aOR: 0.70, 95% CI: 0.59–0.82), as well as lower rates of consistent condom use (aOR: 0.58, 95% CI: 0.51–0.66).

**Conclusion:**

MSMW in Zhejiang Province represent a distinct subgroup with a unique risk profile characterized by gaps in health cognition, specific sexual behavior patterns, and inconsistent condom use. These findings underscore the need for subgroup-specific HIV prevention strategies that move beyond one-size-fits-all approaches and better address the vulnerabilities of MSMW to reduce HIV and STI transmission.

## 1 Introduction

Men who have sex with men (MSM) remain a key high-risk population for HIV, with the epidemic showing a sustained upward trend despite incremental prevention progress (1, 2). Among newly diagnosed HIV infection cases in China in recent years, sexual transmission has predominated, accounting for over 95% of new infections, with MSM accounting for approximately 25% of these sexual transmissions (3). Alarmingly, the HIV infection rates among MSM in certain regions and high-risk subgroups were substantially higher, a trend that highlights the disproportionately elevated vulnerability of these populations (1). Among these heterogeneous groups, men who have sex with men and women (MSMW) has attracted increasing attention as a potential “bridge population” that facilitates HIV transmission between MSM communities and the general heterosexual population (4, 5). Existing evidence indicates that MSMW may differ significantly from men who have sex with men only (MSMO) in terms of sociodemographic background and risky sexual behaviors (6, 7), which in turn affect their susceptibility to HIV and the effectiveness of targeted interventions. For instance, studies in the United States have found that MSMW are more vulnerable to socioeconomic disadvantages, such as higher homelessness rates, and engage in more risk-associated behaviors, such as substance use and commercial sex, than MSMO (8, 9).

Eastern China, particularly the Zhejiang Province, faces a severe HIV burden among MSM, compounded by unique epidemiological characteristics shaped by socioeconomic development, population mobility, and evolving behavioral patterns (10). The province also faces suboptimal uptake of HIV prevention tools; while pre-exposure prophylaxis (PrEP) and post-exposure prophylaxis (PEP) services are available online and offline, their utilization rates among MSM stand at 7.9 and 11.6%, respectively, with inadequate awareness as a key access barrier (11). Previous studies have noted that 40.2% of MSM seek casual partners online and 66.2% engage in unprotected sex with these partners. In comparison, syphilis prevalence reaches 6.5% among HIV-positive MSM—all critical risk markers (12–14). However, most studies treat MSM as a homogeneous group, overlooking the distinctions between MSMO and MSMW.

The subgroup disparities between MSMO and MSMW have become increasingly evident. Among the MSM surveyed in Western China, MSMW constituted 13.7% of the cohort. Notably, this subgroup had significantly higher proportions of low educational levels, non-regular sex partners, unprotected anal intercourse practices, and first-time HIV testing events than MSMO (15). Compared with MSMO, MSMW have lower HIV-related knowledge, higher rates of depression and anxiety, and higher behavioral risks, including inconsistent condom use and significantly greater engagement in commercial and group sex (6, 7, 16). These regional patterns align with global observations, while Asian epidemiological reviews stress the need for localized data to account for cultural and socioeconomic modifiers of risk (9, 17, 18).

Against the backdrop of China’s 2024–2030 AIDS Prevention and Control Plan, which targets 95% coverage of high-risk populations by 2025 and a 10% reduction in high-risk behaviors by 2030 (19), this study aimed to compare the sociodemographic characteristics, health cognition, and risky sexual behaviors of MSMO and MSMW in Zhejiang Province, Eastern China. The findings will clarify subgroup-specific vulnerabilities and inform targeted strategies to achieve national HIV-prevention goals for the MSM population in China.

## 2 Methods

### 2.1 Study design and population

This cross-sectional study was conducted between July and December 2023 in Zhejiang Province, China. Participants were recruited primarily through online channels (including mainstream social media applications and gay-oriented social networking platforms), and convenience sampling was conducted by Non-Governmental Organizations (NGOs) specializing in HIV prevention and sexual health promotion among MSM in Zhejiang Province. The NGOs established digital HIV-prevention service platforms that use location-based Internet services to distribute electronic questionnaires online. All participants could apply for free HIV testing through the platform, with the option of mailed self-testing or in-person testing at an offline service point. To avoid repeated participation by the same individual, we implemented three verification measures: (1) unique questionnaire identification codes for each participant; (2) IP address tracking to exclude duplicate submissions from the same IP; (3) phone number verification for participants applying for free HIV testing, with one phone number corresponding to only one valid questionnaire and one free testing opportunity.

Eligible participants were required to meet the following criteria: (1) biological males aged 16 years or older, (2) reported having had anal or oral sex with at least one man in the past 12 months, (3) resided in the study setting, and (4) provided informed consent electronically. Participants were categorized as MSMW if they reported having engaged in any sexual activity with women in the past 12 months; otherwise, they were classified as MSMO.

### 2.2 Data collection and measures

Data were collected using an anonymous, self-administered online questionnaire hosted on a secure, password-protected survey platform. The questionnaire covered the following domains: (1) Sociodemographic characteristics, including age (≤24, 25–49, or ≥50 years), marital status (single, married/cohabit, or divorced/widowed), education (high school or below, college/university, or master’s degree or above), annual income (<120,000 CNY, or ≥120,000 CNY), and occupation (company employee, student, freelance, or others). (2) Sexual behaviors and identity, including sexual role (top, versatile, bottom), number of sexual partners in the past 3 months (<2, or ≥2), consistent condom use in the past 3 months (never, sometimes use, every time use or no sex), use of sex toys (yes or no), and rush popper use frequency during sex (frequent use, occasional use, or never). (3) Health cognition, including awareness of HIV PrEP (yes or no), PEP (yes or no), and MPOX (yes or no); anxiety about identity affirmation and AIDS phobia (never, mild, moderate, or severe); and awareness of partner’s HIV status (yes or no). (4) HIV/STI prevalence, including HIV status (positive or negative), history of first-time HIV testing (yes or no), and history of STI diagnosis (yes or no).

### 2.3 Statistical analysis

Data were analyzed using SPSS Version 26.0 (IBM Corp., Armonk, NY, USA). No missing data were present for the variables included in the analysis, as the online questionnaire enforced responses for all mandatory items. Descriptive statistics were presented as frequencies and percentages of categorical variables. Chi-square tests were used to compare the sociodemographic characteristics, health cognition, and sexual behaviors of the MSMO and MSMW. A multivariate logistic regression model was used to identify factors associated with MSMW compared to MSMO. All variables with a *p*-value <0.10 in the univariate analysis were included in the multivariate logistic regression model, and the results were expressed as adjusted odds ratios (aORs) with 95% confidence intervals (CIs). All statistical tests were two-sided, and a *p*-value <0.05 was considered statistically significant.

Post-hoc power analysis was performed using PASS 2023 (NCSS, LLC, Kaysville, UT, USA). Based on the observed proportion of MSMW (21.8%, 1665/7629) and the primary outcome of consistent condom use (56.6% in MSMO vs. 37.5% in MSMW; OR: 0.46), with a two-sided *α* = 0.05, the achieved statistical power exceeded 0.999, confirming that the sample size was fully adequate.

## 3 Results

### 3.1 Sociodemographic characteristics and HIV/STI prevalence

This study included 7,629 participants: 5964 with MSMO and 1,665 with MSMW. Participants had a mean age of 28.99 ± 8.54 years. Among them, the median age was 27 years (IQR: 23–32) in the MSMO group and 28 years (IQR: 23–35) in the MSMW group. Overall, most participants were single (5,997/7629, 78.6%), held a college or university degree (5,054/7629, 66.2%), had an annual income of less than 120,000 CNY (6,309/7629, 82.7%), and were employed as company employees (2,843/7629, 37.3%). Additionally, 1.1% (82/7629) tested positive for HIV, 12.9% (982/7629) were undergoing their first HIV test, and 2.6% (201/7629) reported a history of STI. Significant differences were observed between the MSMO and MSMW in terms of age group, marital status, education, income, occupation, and first-time HIV testing. The results are detailed in Table 1.

**Table 1:** The sociodemographic characteristics and HIV/STI status of men who have sex with men and men who have sex with men and women.

Variable	Participants (*n* = 7,629), *n* (%)	MSMO (*n* = 5,964), *n* (%)	MSMW (*n* = 1,665), *n* (%)	*χ*^2^	*p-*value
Age group (years)					
≤24	2,622 (34.4)	2092 (35.1)	530 (31.8)	7.823	0.020
25–49	4,716 (61.8)	3,656 (61.3)	1,060 (63.7)		
≥50	291 (3.8)	216 (3.6)	75 (4.5)		
Marital status					
Single	5,997 (78.6)	4,888 (82.0)	1,109 (66.6)	201.706	<0.001
Married/cohabit	1,388 (18.2)	889 (14.9)	499 (30.0)		
Divorced/widowed	244 (3.2)	187 (3.1)	57 (3.4)		
Education					
High school or below	1851 (24.3)	1,376 (23.1)	475 (28.5)	21.823	<0.001
College/university	5,054 (66.2)	4,022 (67.4)	1,032 (62.0)		
Master’s degree or above	724 (9.5)	566 (9.5)	158 (9.5)		
Income (CNY/year)					
<120,000	6,309 (82.7)	4,959 (83.1)	1,350 (81.1)	3.890	0.049
≥120,000	1,320 (17.3)	1,005 (16.9)	315 (18.9)		
Occupation					
Student	1,254 (16.4)	990 (16.6)	264 (15.9)	11.291	0.010
Company employee	2,843 (37.3)	2,272 (38.1)	571 (34.3)		
Freelance	1,022 (13.4)	782 (13.1)	240 (14.4)		
Others	2,510 (32.9)	1920 (32.2)	590 (35.4)		
HIV status					
Positive	82 (1.1)	67 (1.1)	15 (0.9)	0.606	0.436
Negative	7,547 (98.9)	5,897 (98.9)	1,650 (99.1)		
First-time HIV testing					
Yes	982 (12.9)	679 (11.4)	303 (18.2)	53.875	<0.001
No	6,647 (87.1)	5,285 (88.6)	1,362 (81.8)		
History of STI					
Yes	201 (2.6)	155 (2.6)	46 (2.8)	0.136	0.712
No	7,428 (97.4)	5,809 (97.4)	1,619 (97.2)		

### 3.2 Health cognition and risky sexual behaviors

A comparison of health cognition and risky sexual behaviors between the MSMO and MSMW groups is summarized in Table 2. MSMW reported a higher prevalence of severe anxiety related to identity affirmation and AIDS phobia (Crude odds ratio [cOR]: 1.92, 95% Confidence Interval [CI]: 1.48–2.48). Awareness of PrEP (cOR: 0.51, 95% CI: 0.45–0.57), PEP (cOR: 0.45, 95% CI: 0.40–0.51), and MPOX (cOR: 0.44, 95% CI: 0.39–0.50) was significantly lower among MSMW than MSMO. Regarding sexual behaviors, a substantially higher proportion of MSMW were identified as versatile in their sexual roles (cOR: 11.18, 95% CI: 8.84–14.13). MSMW were less likely to report using sex toys (cOR: 0.49, 95% CI: 0.43–0.55) and consistent condom use (cOR: 0.46 95% CI: 0.41–0.51). Furthermore, MSMW had fewer sexual partners in the preceding 3 months, with 76.5% reporting fewer than two partners compared to 63.9% for MSMO (cOR:1.83, 95% CI: 1.62–2.08). The use of rush poppers was less frequent among MSMW (cOR: 0.38, 95% CI: 0.31–0.46 and cOR: 0.44, 95% CI: 0.37–0.52, respectively). MSMW were also less aware of their partners’ HIV status (cOR: 0.34, 95% CI: 0.30–0.39).

**Table 2:** Comparison of health cognition and risky sexual behaviors between MSMO and MSMW.

Variable	MSMO (*n* = 5,964), *n* (%)	MSMW (*n* = 1,665), *n* (%)	*χ*^2^	*p-*value	cOR (95% CI)	*p-*value
Anxiety about identity affirmation/AIDS phobia						
Severe	175 (2.9)	96 (5.8)	38.139	<0.001	1.92 (1.48–2.48)	<0.001
Moderate	197 (3.3)	45 (2.7)			0.80 (0.57–1.11)	0.184
Mild	1,695 (28.4)	410 (24.6)			0.85 (0.75–0.96)	0.010
Never	3,897 (65.3)	1,114 (66.9)			Reference	
Awareness of PrEP						
Yes	4,719 (79.1)	1,097 (65.9)	125.921	<0.001	0.51 (0.45–0.57)	<0.001
No	1,245 (20.9)	568 (34.1)			Reference	
Awareness of PEP						
Yes	4,855 (81.4)	1,105 (66.4)	172.242	<0.001	0.45 (0.40–0.51)	<0.001
No	1,109 (18.6)	560 (33.6)			Reference	
Awareness of MPOX						
Yes	4,929 (82.6)	1,128 (67.7)	176.593	<0.001	0.44 (0.39–0.50)	<0.001
No	1,035 (17.4)	537 (32.3)			Reference	
Sexual role						
Top	2,203 (36.9)	449 (27.0)	677.490	<0.001	4.20 (3.29–5.37)	<0.001
Versatile	2092 (35.1)	1,135 (68.2)			11.18 (8.84–14.13)	<0.001
Bottom	1,669 (28.0)	81 (4.9)			Reference	
Sex toys use during sexual activity						
Yes	2,204 (37.0)	370 (22.2)	126.375	<0.001	0.49 (0.43–0.55)	<0.001
No	3,760 (63.0)	1,295 (77.8)			Reference	
Consistent condom use in the past 3 months						
Yes	3,375 (56.6)	624 (37.5)	190.624	<0.001	0.46 (0.41–0.51)	<0.001
No	2,589 (43.4)	1,041 (62.5)			Reference	
Number of sexual partners in the past 3 months						
<2	3,813 (63.9)	1,273 (76.5)	91.855	<0.001	1.83 (1.62–2.08)	<0.001
≥2	2,151 (36.1)	392 (23.5)			Reference	
Rush poppers use during sex activity						
Frequently use	943 (15.8)	125 (7.5)	173.257	<0.001	0.38 (0.31–0.46)	<0.001
Occasional use	1,137 (19.1)	175 (10.5)			0.44 (0.37–0.52)	<0.001
Never	3,884 (65.1)	1,365 (82.0)			Reference	
Awareness of partner’s HIV status						
Yes	2,852 (47.8)	599 (36.0)	284.131	<0.001	0.34 (0.30–0.39)	<0.001
Unsure	2,197 (36.8)	504 (30.3)			0.37 (0.32–0.43)	<0.001
No	915 (15.3)	562 (33.8)			Reference	

Percentages may not add up to 100% due to rounding.

### 3.3 Multivariable logistic regression analysis of factors associated with MSMW compared to MSMO

**Figure 1:**
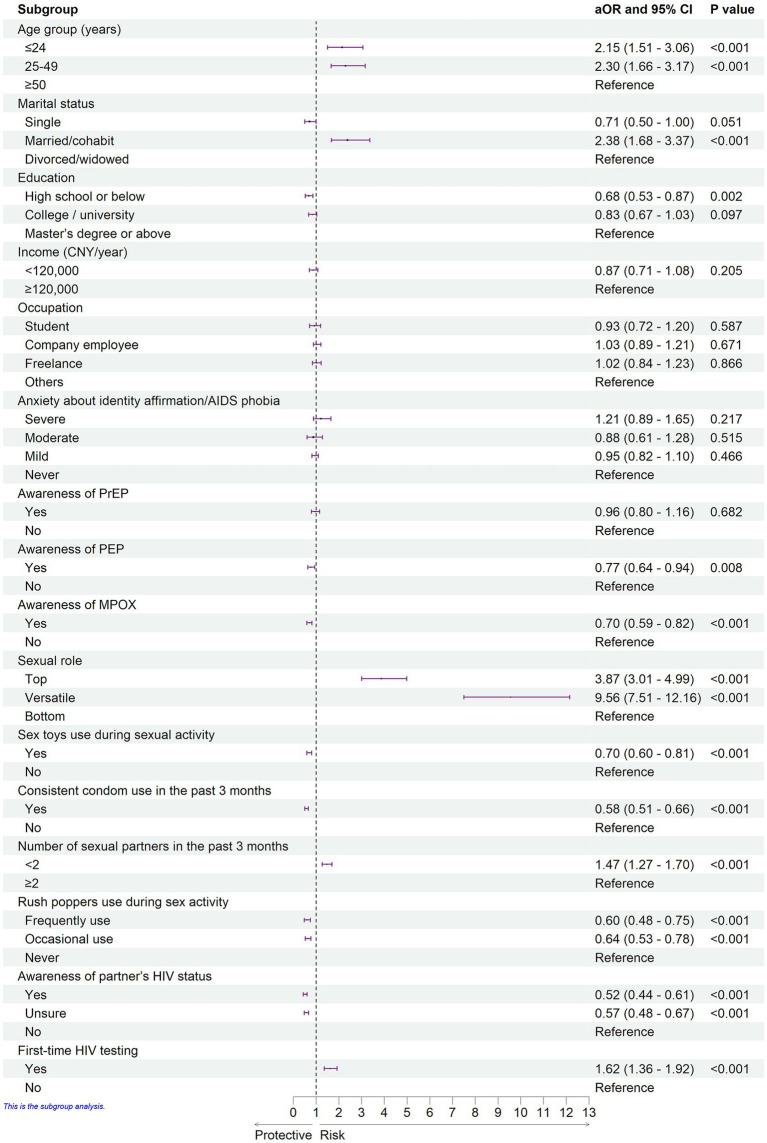
Adjusted odds ratios for factors associated with MSMW compared to MSMO.

The results of the multivariate logistic regression model identifying factors associated with MSMW compared with MSMO are presented in Figure 1. Younger age was significantly associated with a higher odds of MSMW. Participants aged ≤24 years and 25–49 years had higher odds of MSMW than those aged ≥50 years (adjusted odds ratio [aOR]: 2.15, 95% CI: 1.51–3.06 and aOR: 2.30, 95% CI: 1.66–3.17, respectively). Marital status was a strong predictor; married or cohabiting individuals had significantly higher odds of MSMW than their divorced or widowed counterparts (aOR: 2.38, 95% CI: 1.68–3.37). Compared with participants with a master’s degree or higher, those with a high school education or below had lower odds of MSMW (aOR: 0.68, 95% CI: 0.53–0.87).

Health cognitive factors also have a significant effect. Awareness of PEP (aOR: 0.77, 95% CI: 0.64–0.94) and MPOX (aOR: 0.70, 95% CI: 0.59–0.82) were independently associated with lower odds of MSMW. Sexual behavioral patterns were a strong predictor of MSMW. Relative to the ‘bottom’ sexual role, participants who identified as ‘top’ (aOR: 3.87, 95% CI: 3.01–4.99) or ‘versatile’ (aOR: 9.56, 95% CI: 7.51–12.16) had markedly higher odds of MSMW. Sex toy use (aOR: 0.70, 95% CI: 0.60–0.81) and consistent condom use (aOR: 0.58, 95% CI: 0.51–0.66) were negatively associated with MSMW. Having fewer than two sexual partners was associated with higher odds of MSMW (aOR, 1.47; 95% CI: 1.27–1.70). Both frequent and occasional use of rush poppers correlated with lower odds of MSMW (aOR: 0.60, 95% CI: 0.48–0.75 and aOR: 0.64, 95% CI: 0.53–0.78, respectively). In addition, MSMW had lower odds of being aware of their partners’ HIV status (aOR: 0.52, 95% CI: 0.44–0.61). Notably, first-time HIV testing was associated with higher odds of MSMW (aOR: 1.62, 95% CI: 1.36–1.92).

## 4 Discussion

This study provides a comprehensive comparison of the sociodemographic profiles, health cognition, risky sexual behaviors, and healthcare engagement between MSMO and MSMW in China. The findings reveal profound and systematic disparities, painting a complex picture of vulnerability specific to MSMW, and underscoring the critical need to move beyond a homogenized view of the MSM population for effective public health strategies.

Our analysis confirmed that MSMW constitutes a distinct demographic subgroup. They were significantly older and had a much higher likelihood of being married or cohabiting, which is consistent with findings from both Eastern and Western China (6, 7). Marital status is a defining and risk-enhancing characteristic, often reflecting compliance with profound sociocultural and familial pressures in China, which force many MSM into heterosexual marriages or relationships (20, 21). This “double life” creates a unique stressor, directly reflected in our finding that MSMW report a significantly higher prevalence of severe anxiety related to identity affirmation or AIDS phobia compared to MSMO. This mental health burden is a critical public health concern as studies have consistently linked depression and anxiety among MSM to increased sexual risk-taking, substance use, and poorer healthcare engagement (22–24). Lower educational attainment was inversely associated with MSMW. This may reflect the generally higher educational attainment in eastern China, which affords MSMW greater access to social resources yet does not resolve their knowledge deficits regarding HIV/STI prevention.

A pivotal and alarming finding is the significant “prevention gap” in health cognition among MSMW. Their awareness of the key biomedical prevention tools, PrEP, PEP, and MPOX, was substantially lower than that of the MSMO. Multivariate analysis confirmed that the awareness of PEP and MPOX was independently associated with lower odds of MSMW. This indicates that knowledge of these critical tools is strongly concentrated within the more connected MSMO community, indicating a structural failure in health information dissemination (25). Currently, HIV/STI prevention campaigns in China are often delivered through MSM-focused community-based organizations, gay social networking apps, and sexual health clinics frequented by MSM. MSMW, who may be less integrated into visible gay social circles or avoid MSM-specific venues because of stigma or marital obligations, are systematically missed by these channels, creating profound information inequity that denies them access to the knowledge required for modern, effective self-protection (26).

The sexual behavior profile of the MSMW is complex and reveals its potential role as a “bridge” for HIV/STI transmission. The most striking behavioral factor is sexual role (27). MSMW had dramatically higher odds of identifying as “versatile” or “top” compared to exclusively “bottom.” Versatility biologically increases the efficiency of potential transmission across sexual networks (28). This role, combined with sexual contact with women, positions MSMW at a critical intersection between MSM and heterosexual networks. Other behavioral findings seem contradictory. On the one hand, MSMW reported having fewer male sexual partners in the preceding 3 months and were less likely to use rush poppers, factors typically associated with lower risk in MSM epidemiology (29). On the other hand, and of paramount concern, they reported significantly lower rates of consistent condom use (30). The multivariable model powerfully underscored this finding, showing that consistent condom use was strongly associated with lower odds of being an MSMW. This suggests that the inconsistent or non-use of condoms is a defining and predominant risk behavior in this group. A lower number of partners does not mitigate the high per-act risk introduced by inconsistent condom use, especially when coupled with low awareness of partners’ HIV status. This pattern may be driven by the dynamics in their relationships with women, which differ from the norms of many casual MSMO partnerships (31). Furthermore, our previous study highlighted that MSMW were more likely to seek casual partners online and offline and engage in commercial and group sex (16), adding layers to their risk environment, although the study showed lower partner numbers.

Analysis of healthcare engagement yielded nuanced insights. The significant association between first-time HIV testing and MSMW is particularly important. This finding is consistent with previous studies (32, 33). This suggests that a significant proportion of MSMW are tested only after reaching a point of perceived risk or concern, rather than engaging in regular, preventive screening. This “first-test” moment represents a critical window of opportunity for intervention—to provide comprehensive education, link to prevention tools like PrEP, and offer counseling. However, this potential opportunity is tempered by broader patterns of disengagement. Low overall awareness of prevention tools is a major barrier. Furthermore, the lower use of sex toys, which can be associated with certain MSM subcultures and safer sex practices, may indicate a more general disconnection from the harm-reduction discourse that is prevalent in some MSMO communities.

This study has several limitations. The cross-sectional design of this study precludes causal inferences. Data on sensitive behaviors were self-reported and subject to social desirability bias. Although large-scale recruitment may lead to underrepresentation of the most hidden MSMW, our definition of MSMW does not capture the frequency or context of encounters with women, which may further influence risk profiles. Future research should employ longitudinal designs to establish causality, particularly between mental health states and risky behaviors. Mixed-method studies are crucial for understanding the lived experiences and specific barriers faced by MSMW. Intervention research must develop and test targeted strategies to reach MSMW, potentially through online platforms, not exclusively gay-identified general healthcare settings or discreet, destigmatized service delivery models.

## 5 Conclusion

In conclusion, this study provides robust evidence that MSMW in Zhejiang Province, Eastern China represent a distinct subgroup with a unique risk profile characterized by significant gaps in health cognition, specific sexual behavior patterns such as versatile roles, inconsistent condom use, and lower engagement with HIV testing services. The identified factors, including marital status, awareness of preventive tools, and condom use practices, underscore the need to move beyond one-size-fits-all interventions to a broader MSM population. Future public health strategies must be tailored to address the specific sociobehavioral and cognitive needs of MSMW. Such targeted efforts are not only crucial for safeguarding their health. Still, they are also an essential component of a comprehensive approach to interrupt the transmission of HIV and other STI within and beyond their sexual networks.

## Data Availability

The raw data supporting the conclusions of this article will be made available by the authors, without undue reservation.
